# Feasibility Randomised Controlled Trial of a New Digital Intervention (‘FRAME’) to Promote Resilience in Women Treated for Primary Breast Cancer

**DOI:** 10.1002/pon.70217

**Published:** 2025-07-09

**Authors:** Anna V. Cartwright, Hannah Krzyzanowski, Rona Moss‐Morris, Laura Smith, Yogini Sawjani, Camilla Böhme Kristensen, Nuvera Mukaty, Sam Norton, Jo Armes, Colette R. Hirsch

**Affiliations:** ^1^ Institute of Psychiatry Psychology and Neuroscience King's College London London UK; ^2^ Faculty of Dentistry Oral & Craniofacial Sciences King's College London London UK; ^3^ Faculty of Health and Medical Sciences School of Health Sciences University of Surrey Guildford UK; ^4^ South London and Maudsley NHS Foundation Trust London UK

**Keywords:** bias, breast cancer, cancer survivors, cognitive bias modification, interpretation bias, positive psychology, psycho‐oncology, psychological, resilience, survivorship

## Abstract

**Objective:**

This study investigates the acceptability of a novel Cognitive Bias Modification for Interpretation (CBM‐I) intervention, ‘FRAME’, to promote resilience in women who have completed active treatment for primary breast cancer and determines the feasibility of a full‐scale randomised controlled trial.

**Methods:**

A two‐armed, participant‐blind, parallel groups randomised controlled trial of CBM‐I versus a time‐matched control. Participants were recruited from community organisations and social media. Measures of acceptability, feasibility, change in interpretation bias and clinical outcomes (resilience, mood and quality of life) were assessed at baseline (T0), 1‐month post‐randomisation (T1, end of intervention), 2‐month (T2) and 4‐month post‐randomisation (T3).

**Results:**

Sixty‐seven participants completed baseline assessment and were randomised to the FRAME CBM‐I (*n* = 35) or control group (*n* = 32). Acceptability of CBM‐I met pre‐specified progression criteria, and 80% adhered to the CBM‐I intervention. Between‐group differences in interpretation bias at T1 demonstrated a moderate effect in favour of CBM‐I on two measures of interpretation bias (SMD_g_ = 0.66 and 0.73). Effect size estimates suggest moderate treatment effects on resilience (SMD_g_ = 0.64) and small effects on mood, in favour of FRAME. No intervention‐related adverse events were reported.

**Conclusions:**

The study results provide strong support for the acceptability of a new online CBM‐I intervention (‘FRAME’) to promote resilience in women treated for primary breast cancer and indicate that a full‐scale trial is feasible. The study fulfiled all pre‐specified progression criteria to advance to an efficacy trial, except meeting the recruitment target of 70 participants. Importantly, recruitment took place during the Covid‐19 pandemic. Recommendations for future research are provided.

## 1 Background

Female breast cancer (BC) is the most diagnosed cancer worldwide [1]. In the UK, one in 7 women develop BC [2], which often leads to significant physical, emotional, social, and practical challenges for individuals and their loved ones [3, 4]. Consequently, psychological distress amongst people living with and beyond BC is common [5].

### 1.1 Breast Cancer and Resilience

Psycho‐oncology research often focusses on adverse impacts of cancer. However, most people diagnosed with primary BC adapt and adjust, with many following ‘resilient’ trajectories into survivorship [6]. Amongst BC survivors, greater emotional resilience (i.e. ability to adapt and cope with adversity) has been associated with lower anxiety, depression, fear of recurrence and worry, and greater quality of life [7, 8]. Thus, increasing resilience may have a range of positive impacts [9].

Psychosocial interventions targeting resilience have been developed [10]. However, meta‐analyses highlight that many studies are of low quality, and existing interventions are predominantly delivered face‐to‐face or in groups [11, 12]. Due to the large number of people affected by BC and limited clinical resource, therapist‐delivered care cannot meet demand, with approximately 45%–75% of BC survivors reporting unmet supportive care needs [13]. Utilising technology may improve access to care [14]. However, challenges of suboptimal engagement affecting digitally delivered interventions highlight the need to maximise acceptability [15, 16].

### 1.2 Cognitive Processes and Resilience

Developing interventions tailored to the specific needs of a population is central to enhancing engagement. Furthermore, to ensure novel interventions are likely effective, it is crucial to target key mechanisms of change [17]. The cognitive model of resilience identifies three cognitive processes that may maintain resilience: adaptive cognitive biases, active positive cognitions and executive control processes [18]. Research indicates that individuals who have cancer compared to those who do not, are more likely to interpret ambiguous information as health related [19, 20]. Crucially, research has suggested that these biases are associated with persistent distress following breast cancer [21]. An experimental study investigating attention and interpretation biases and executive control in women after BC treatment found that the tendency to interpret ambiguous information in a more negative, cancer‐related fashion predicted lower self‐reported resilience 6 months later [7]. Interpretation bias may thus be a promising intervention target for promoting resilience.

Cognitive Bias Modification interventions, believed to modify relatively automatic processes [22], are well‐suited to remote delivery. Numerous multi‐session online interpretation training programmes have been developed to change interpretation biases [23]. One type of Cognitive Bias Modification for Interpretation (CBM‐I) training has been found to reduce worry, anxiety, and depression [24, 25]. This involves participants listening to scenarios about common situations, and answering questions about them, with feedback encouraging positive interpretations [26]. We have adapted this approach by tailoring training materials to address resilience after BC treatment, using ambiguous scenarios commonly experienced by BC survivors, such as coping with side‐effects and changes in appearance.

### 1.3 Current Study

The aims were to: evaluate the acceptability of a new CBM‐I intervention (‘Finding Resilient Answers More Effectively’, ‘FRAME’) to foster resilience in women after active treatment for primary BC, and who score below average on resilience; explore whether FRAME reduces cancer‐specific negative interpretation bias and increases resilience, compared with a time‐matched control; and assess the feasibility of a full‐scale randomised controlled trial. Objectives were to: assess acceptability of and adherence to the intervention; determine rates of recruitment and retention; test the utility of outcome measures; and explore mechanistic change and treatment effects.

## 2 Methods

### 2.1 Design

Two‐armed (CBM‐I; control), participant‐blind, parallel groups randomised controlled trial. Assessments took place at: baseline/pre‐randomisation (T0) and 1‐month (T1), 2‐month (T2) and 4‐month (T3) post‐randomisation.

### 2.2 Study Registration and Approval

The study was preregistered with Open Science Framework (https://doi.org/10.17605/OSF.IO/HTR3Z) and approved by King's College London ethics committee (REF: HR‐19/20‐14437). Informed consent was obtained. Progression criteria were specified prior to analysis (Supporting Information S1: Supplementary materials 1). During recruitment, amendments were made to inclusion criteria, given challenges posed by the Covid‐19 pandemic (maximum time since finishing treatment changed from 2 to 3 years), and to reflect Generalised Anxiety Disorder Scale (GAD‐7; [27]) and Patient Health Questionnaire‐9 (PHQ‐9; [28]) clinical cut‐offs (changed from > 8 to ≥ 8 and from > 8 to ≥ 10, respectively). Measures of quality of life and psychological distress were added following preregistration.

### 2.3 Participants

A target sample of 70 randomised participants was set, based on the rule of thumb for feasibility trials to include at least 30 participants per arm and enable sample size calculation for a larger trial [29].

Inclusion criteria were: female; aged 18–85 years; diagnosed with primary BC; completed active cancer treatment 4 months—3 years previously; access to the internet and email; able to use a computer independently; normal or corrected‐to‐normal vision and hearing; can read and understand English; and score ≤ 68 on the Connor‐Davidson Resilience Scale [30], ≥ 8 on the GAD‐7 and ≥ 10 on the PHQ‐9.

Exclusion criteria were: diagnosis of stage 4 cancer; currently has any other cancer diagnosis; residing outside the UK; not registered with a UK general practitioner; comorbid psychosis, bipolar disorder, personality disorder or substance abuse; past or current risk to self (score > 1 on PHQ‐9 item 9, suicide attempt in the past 2 years, self‐harm in the past 12 months, and/or indicate suicidal ideation); severe depression (≥ 23 on PHQ‐9); started or changed psychiatric medication within past 6 weeks; previously taken part in university studies measuring interpretation bias.

### 2.4 Interventions

#### 2.4.1 CBM‐I

CBM‐I intends to train participants to process ambiguity in a more benign way. A CBM‐I training, effective in reducing worry, rumination, depression and anxiety [24, 25], was adapted using novel training materials, developed by conducting a series of interviews with women who had lived experience of BC (see Supporting Information S1: Supplementary materials 2 for examples), combined with scenarios relating to worry and rumination, as used by Hirsch and colleagues [24]. Piloting of a sample of the novel CBM‐I materials indicated they were ambiguous and relevant to BC survivors [31].

Over 3‐weeks, participants completed 10 training assignments (each 25–30 min) via a purpose‐built website, comprising 300 total scenarios (‘trials’). As with previous studies [24, 25], the first assignment began with brief imagery training. During assignments, participants heard ambiguous audio scenarios and vividly imagined themselves as the central character. Scenarios were resolved positively (20 scenarios, e.g. 'It is New Year's Day, and you find yourself reflecting on how things have changed over the years. You think about what life will bring in the next year and feel hopeful’) or remained unresolved (10 novel, 10 repeated from positively resolved scenarios but with ambiguity remaining), whereby participants imagined their own positive/benign ending (e.g. ‘[…] You think about the prospect of what life will bring in the next year’). Participants rated positivity (50% of trials) or vividness (50% of trials) of their image, using a visual analogue scale (0 = not at all to 10 = extremely). Feedback reinforced vivid imagery and positive/benign interpretations. Following each scenario, participants completed a ‘comprehension’ question (yes/no), relating to resolution of ambiguity (e.g. ‘Do you feel optimistic/pessimistic thinking about the next year?’). Accuracy feedback was provided (for unresolved scenarios feedback was for correct responses only).

#### 2.4.2 Time‐Matched Control

Participants heard 50 scenarios per assignment for 10 assignments, with 400 unique scenarios. Ambiguity remained unresolved, with no instructions to resolve ambiguity or imagine positive endings. The first assignment began with a time‐matched comprehension filler task (see [24]). Half the scenarios were followed with a comprehension (yes/no) question relating to ambiguity, but without feedback. The remainder were followed by factual (yes/no) questions, with accuracy feedback provided for incorrect responses. Participants did not rate positivity or vividness.

### 2.5 Randomisation and Blinding

Participants were randomised using simple randomisation in a 1:1 allocation ratio after completing T0. Before recruitment, a random allocation sequence was generated using https://www.random.org by a researcher independent of the study team. The sequence was concealed from researchers involved in recruitment and data collection to minimise risk of bias. Upon recruitment, the researcher contacted an administrator, not otherwise involved in running the study, to request randomisation. The administrator looked up the allocation sequence and informed the researcher of the allocation condition. Participants, but not researchers, were blind to allocation until after T3.

### 2.6 Measures

#### 2.6.1 Feasibility

Number of people who registered on the platform. The number and proportion of those registered who were screened; those screened who were eligible; and those eligible who enroled. Retention to assessment and reasons for exclusion and drop‐out were recorded.

#### 2.6.2 Expectancy and Acceptability

Expectancy (T0): participants rated how logical the programme seemed and how useful they thought it will be. Acceptability (T1): participants rated how logical and useful the programme was and their confidence recommending it to a friend. Items were measured on a five‐point scale from 0 ‘not at all logical/useful/confident’ to 4 ‘very/extremely logical/useful/confident’ (see Supporting Information S1: Supplementary materials 3).

#### 2.6.3 Adherence

Assignments were considered complete if participants completed ≥ 50% of trials. Adherence was classified as ‘full’ (8–10 assignments), ‘partial’ (1–7 assignments) or ‘no intervention’ (0 assignments).

#### 2.6.4 Self‐Report Outcomes

##### 2.6.4.1 Resilience

Resilience (past month) was measured using the 25‐item Connor Davidson Resilience Scale (CD‐RISC, [30]). Items, rated on a 5‐point Likert scale from 0 (not true at all) to 4 (true nearly all of the time), are summed (0–100). Higher scores indicate higher resilience.

##### 2.6.4.2 Anxiety

Anxiety symptoms (past 2 weeks) were assessed using the seven‐item GAD‐7 [27]. Items, rated on a 4‐point Likert scale from 0 (not at all) to 3 (nearly every day), are summed (range: 0–21). Higher scores indicate more severe anxiety. Cut‐offs of 5, 10, and 15 indicate mild, moderate and severe anxiety [27].

##### 2.6.4.3 Depression

Depressive symptoms (past 2 weeks) were assessed using the nine‐item PHQ‐9 [28]. Items, rated on a 4‐point Likert scale from 0 (not at all) to 3 (nearly every day), are summed (range: 0–27). Higher scores indicate more severe depression. Cut‐offs of 5, 10, 15 and 20 indicate mild, moderate, moderately severe and severe depression [28].

##### 2.6.4.4 Psychological Distress

The 15‐item Patient Health Questionnaire‐Anxiety and Depression Scale (PHQ‐ADS), measuring psychological distress [32], is a composite of the PHQ‐8 and GAD‐7. Items are summed (range: 0–45). Cut‐offs of 10, 20 and 30 indicate mild, moderate and severe distress [33].

##### 2.6.4.5 Quality of Life (QoL)

The European Organisation for Research and Treatment of Cancer QoL Questionnaire for Cancer Patients (EORTC QLQ‐C30; [34]) Version 3 measures health‐related QoL. There are five functional scales (physical, role, cognitive, emotional, and social functioning), three symptom scales (fatigue, nausea and vomiting, and pain), a global health status/QoL scale and six single items (appetite loss, diarrhoea, dyspnoea, constipation, insomnia, financial impact). Scores are linearly converted (range: 0–100). Higher scores indicate more symptom burden (symptom scales) or better health (functional and global scales). Summary scores are calculated as the mean of 13 subscales (excluding global QoL and financial impact), with symptom scores reversed. Higher summary scores indicate greater QoL.

The 23‐item European Organisation for Research and Treatment of Cancer QoL Questionnaire for Breast Cancer Patients (EORTC QLQ‐BR23; [35]) measures aspects of QoL specific to BC. Five multi‐item scales assess systemic therapy side‐effects, arm symptoms, breast symptoms, body image and sexual functioning. Single‐item scales measure upset by hair loss (if applicable), future perspective, and sexual enjoyment (if applicable). Scales range from 0 to 100. Higher scores represent healthier functioning (functional scales) or more severe symptomatology (symptom scales).

##### 2.6.4.6 Interpretation Bias

###### 2.6.4.6.1 Recognition Test (RT)

Two versions of the RT [36] were generated, adapted for BC survivors [7], counterbalanced across participants at T0 and T1. Participants read 12 ambiguous scenarios (six BC specific, four worry‐related, two depression‐related), whilst imagining themselves as the central character. Comprehension questions checked encoding and understanding. Participants were later presented with scenario titles and four statements: two ‘targets’ which resolved ambiguity (positive/negative), and two ‘foils’ (positive/negative). Participants rated how similar in meaning each sentence was to the scenario on a four‐point scale from 1 (very different) to 4 (very similar). A RT Index was calculated by subtracting the mean similarity ratings for negative targets from mean ratings for positive targets. Higher scores indicate more positive bias.

###### 2.6.4.6.2 Scrambled Sentences Task (SST)

Participants use five of six presented words to create a grammatically correct sentence, whilst holding a string of six digits in mind [37]. Sentences can be negatively or positively valanced. Participants unscrambled up to 20 items (50% worry‐related [38], 50% depressive rumination‐related [37]) in 5‐minutes, then recalled the digit string. Two lists were counterbalanced across participants over T0 and T1. The number of positively unscrambled sentences divided by the total number of grammatically correct sentences provides an interpretation bias index (range: 0–1). Higher scores indicate more positive bias.

### 2.7 Procedure

Participants were recruited via social media and community organisations by accessing the online ‘FRAME’ platform, containing information and screening consent. The information sheet said that the intervention aims to ‘reduce worry and low mood by building new thinking habits that promote resilience’. They completed online screening and a telephone call to determine suitability. Once enroled, participants completed baseline measures (T0)—including interpretation bias measures, self‐report questionnaires and demographic and clinical questions—within a few days of enrolment. They then received information about the interventions, completed expectancy measures, were randomised, and completed the first intervention assignment the same day.

Participants were encouraged to complete three assignments per week, completing all assignments over 3‐week (possible extension to 1‐month). Researchers checked understanding after the first assignment. Automatic email reminders encouraged timely completion. Emails/texts after assignments 5 and 7 encouraged application of techniques in daily life (CBM‐I) or thanked them for participating (control). Researchers monitored adherence and contacted participants to encourage completion, if required.

At T1, participants completed post‐training measures, comprising the same as T0 (excluding demographic and clinical characteristics). Approximately 1‐week later, 47 participants, purposively selected to include diversity of ages, ethnicities and intervention condition, were invited to a 30–45 min feedback interview (reported elsewhere). At T2 and T3, participants completed follow‐up questionnaires online via ‘Qualtrics’.

### 2.8 Adverse Events and Intercurrent Events

A record of possible confounding variables and adverse events was updated throughout the study. At assessments, participants were asked whether: they had deliberately harmed themselves, attempted to take their life or attended hospital for reasons related to mental health; symptoms had changed (if yes, reasons for this); mental health treatments had changed; and/or they had experienced negative life events. Potential adverse events were coded on a five‐point scale as to whether they were study‐related (‘unrelated’, ‘unlikely’, ‘possibly’, ‘likely’, or ‘definitely’) and considered adverse events when rated as ‘definitely’ or ‘likely’ due to the intervention. Where necessary, participants were contacted for additional information.

### 2.9 Statistical Analysis

Statistical analysis was conducted using IBM SPSS version 29 and Jamovi. Descriptive statistics summarised sample characteristics, recruitment and retention rates and adherence. Binomial exact confidence intervals are reported.

Progression criteria (‘green’ range) were defined as: 100% target sample recruited; > 70% retention (T3); > 70% intervention adherence; average score > 2 on acceptability measures (logic and usefulness, T1); upper bound of Hedge's *g* effect size > 0.5 for change in interpretation bias; no serious adverse events attributed to CBM‐I. For ‘amber’ and ‘red’ criteria, see Supporting Information S1: Table S1.

Outcome measure scores were plotted to determine skew and kurtosis. Floor and ceiling effects at T0 and T1 were considered significant if more than 15% of respondents achieved minimum/maximum scores. Internal consistency was assessed using Cronbach's alpha coefficient (T0). Means and standard deviations (SD) are provided for self‐report outcomes and interpretation bias measures.

Interpretation bias treatment effect (mechanistic outcome) was estimated using linear regression models, including post‐intervention assessment of interpretation bias as the outcome variable. Treatment arm was included as a dummy coded variable and baseline level of the outcome as a covariate. Treatment effects on self‐report measures were estimated using linear mixed effects models, with a random effect to account for repeated measures within participants over time. Treatment arm and time were included as dummy coded variables, and arm × time interaction terms. Baseline levels of outcomes were included as covariates. Mean differences, confidence intervals (CI) of mean differences, effect sizes (Hedge's *g* based on the observed variance) and confidence intervals of effect sizes are reported. As a feasibility trial, the sample size is not powered to detect between group differences, so statistical significance is not reported. Effects are interpreted with respect to the 95%CIs. Additional sensitivity analysis was conducted with those retained to T1, who completed ≥ 8 assignments (see Supporting Information S1: Table S4).

## 3 Results

### 3.1 Participant Flow and Recruitment

**FIGURE 1:**
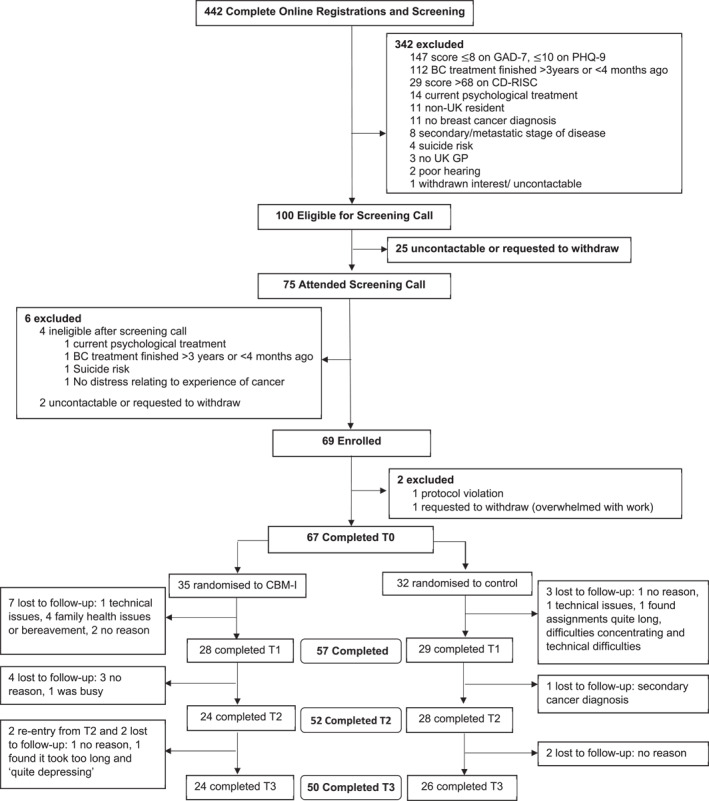
Participant flow diagram.

Figure 1 presents participant flow. From 29 June 2020 to 27 November 2021, 442 individuals registered and completed online screening, of which 75 attended telephone screening (17.0%, 95%CI 13.6–20.8). An additional 142 incomplete questionnaires were submitted (45 duplicates). Of those attending telephone screening, 71/75 were eligible (94.7%, 95%CI 86.9–98.5) and 69/71 enroled (97.2%, 95% CI 91.2–99.7). One protocol violation occurred (participant randomised before T0) and was excluded from analyses. The final sample (*N* = 67) is below the pre‐specified target (*N* = 70).

### 3.2 Baseline Characteristics

Baseline demographic and clinical characteristics are displayed in Table 1. Fifteen participants finished active treatment > 24 months before T0 (maximum = 30 months).

**TABLE 1:** Summary of baseline demographic and clinical characteristics, by intervention group.

Characteristic	CBM‐I		Control	
	*N*		*N*	
Age in years, median (IQR)	35	50 (9)	32	48 (14)
Education, *n* (%)	35		32	
Secondary		8 (22.9)		4 (12.5)
Bachelors		13 (37.1)		9 (28.1)
Masters		6 (17.1)		11 (34.4)
Doctoral		2 (5.7)		3 (9.4)
Other		6 (17.1)		5 (15.6)
Marital status, *n* (%)	35		32	
Single, never married		9 (25.7)		8 (25.0)
Married		20 (57.1)		21 (65.6)
Divorced		6 (17.1)		3 (9.4)
Ethnic group, *n* (%)	35		32	
White		34 (97.1)		30 (93.8)
Other		1 (2.9)		2 (6.2)
Nationality, *n* (%)	35		32	
British		32 (91.4)		28 (87.5)
European		2 (5.7)		3 (9.4)
Other		1 (2.9)		1 (3.1)
Time since finishing active cancer treatment in months, median (IQR)	33	17 (16)	31	13 (17)
Previous treatments *n* (%)	33		32	
Surgery only		1 (3.0)		5 (15.6)
Chemotherapy only		0 (0)		0 (0)
Radiotherapy only		5 (15.2)		2 (6.3)
Surgery/Chemotherapy		3 (9.1)		2 (6.3)
Surgery/Radiotherapy		4 (12.1)		6 (18.8)
Chemotherapy/Radiotherapy		2 (6.1)		1 (3.1)
Surgery/Radiotherapy/Chemotherapy		18 (54.5)		16 (50.0)
Past psychological treatment, *n* (%)	33	25 (71.4)		21 (65.6)
Current psychological treatment, *n* (%)	33	6 (18.2)	31	12 (38.7)
Past psychotropic medication, *n* (%)	33	14 (42.4)	31	9 (29.0)
Current psychotropic medication, *n* (%)	33	8 (24.2)	31	11 (35.5)

Abbreviations: CBM‐I, Cognitive Bias Modification for Interpretation; IQR, Interquartile Range.

### 3.3 Retention and Adherence

Logistic regression analysis indicated that no demographic or clinical characteristics were associated with missingness at T1. Table 2 shows retention and adherence to assignments. Retention to T3 in the total sample (74.6%, 95% CI 62.5–84.5) and full adherence to CBM‐I (80.0%, 95% CI 63.1–91.6) were in the ‘green’ range on progression criteria (> 70%).

**TABLE 2:** Retention and adherence.

	Total sample (*N* = 67)	CBM‐I (*N* = 35)	Control (*N* = 32)
	*N* (% [95% CI])	*N* (% [95% CI])	*N* (% [95% CI])
Assessments completed			
T1	57 (85.1, [74.3–92.6])	28 (80.0 [63.1–91.6])	29 (90.6 [75.0–98.0])
T2	52 (77.6, [65.8–86.9])	24 (68.6 [50.7–83.1])	28 (87.5 [71.0–96.5])
T3	50 (74.6, [62.5–84.5])	24 (68.6 [50.7–83.1])	26 (81.2 [63.6–92.8])
Adherence to assignments			
Full	53 (79.1 [67.4–88.1])	28 (80.0 [63.1–91.6])	25 (78.1 [60.0–90.7])
Partial	10 (14.9 [7.4–25.7])	5 (14.3 [4.8–30.3])	5 (15.6 [5.3–32.8])
None	4 (6.0 [1.7–14.6])	2 (5.7 [0.7–19.2])	2 (6.3 [0.8–18.4])

Abbreviations: CBM‐I, Cognitive Bias Modification for Interpretation; CI, Confidence Interval.

### 3.4 Expectancy and Acceptability

The possible range on expectancy and acceptability questions was 0 (not at all) to 4 (very/extremely). T0 median logic ratings were 3 (both groups) and usefulness ratings were 2 (control) and 3 (CBM‐I). T1 median logic ratings were 3 (both groups) and usefulness ratings 1 (control) and 3 (CBM‐I). Therefore, T1 acceptability ratings (CBM‐I) were in the ‘green’ range (> 2) on pre‐specified criteria. Median scores for recommending the programme were 1 (control) and 3 (CBM‐I). For frequencies and percentages, see Supporting Information S1: Tables S2 and S3.

### 3.5 Reliability and Responsiveness of Self‐Report Outcome Measures

At T0 and T1, there were no missing items on measures. Range, floor and ceiling effects are presented (Table 3).

**TABLE 3:** Reliability and responsiveness of self‐report outcome measures.

Measure	Possible range	Observed range		% at floor		% at ceiling		α
		T0	T1	T0 (*N* = 67)	T1 (*N* = 57)	T0 (*N* = 67)	T1 (*N* = 57)	T0
CDRISC	0–100	27–77	34–94	0	0	0	0	0.87
PHQ‐9	0–27	2–21	1–21	0	0	0	0	0.73
GAD‐7	0–21	1–21	0–21	0	1.8	1.5	1.8	0.85
PHQ‐ADS	0–45	4–39	3–42	0	0	0	0	0.86
EORTC QLQ‐30								
Physical functioning	0–100	40.00–100	40.00–100	0	0	22.4^a^	28.1^a^	0.75
Role functioning	0–100	0–100	0–100	3.0	3.5	26.9^a^	28.1^a^	0.75
Cognitive functioning	0–100	0–100	0–100	4.5	5.3	10.4	8.8	0.67
Emotional functioning	0–100	0–83.33	0–100	3.0	1.8	0	1.8	0.71
Social functioning	0–100	0–100	0–100	13.4	3.5	16.4^a^	26.3^a^	0.88
Fatigue	0–100	0–100	0–88.89	1.5	1.8	4.5	0	0.81
Nausea and vomiting	0–100	0–100	0–50.00	68.7^a^	66.7^a^	4.5	0	0.50
Pain	0–100	0–100	0–100	14.9	12.3	7.5	3.5	0.86
Appetite loss	0–100	0–100	0–100	80.6^a^	78.9^a^	1.5	1.8	N/A
Diarrhoea	0–100	0–100	0–66.67	71.6^a^	80.7^a^	1.5	0	N/A
Dyspnoea	0–100	0–100	0–66.67	56.7^a^	57.9^a^	1.5	0	N/A
Constipation	0–100	0–100	0–66.67	68.7^a^	71.9^a^	3.0	0	N/A
Insomnia	0–100	0–100	0–100	4.5	17.5^a^	25.4^a^	17.5^a^	N/A
Financial impact	0–100	0–100	0–66.67	55.2^a^	64.9^a^	3.0	0	N/A
Global health status	0–100	8.33–75.00	16.67–83.33	0	0	0	0	0.67
Summary score	0–100	32.99–91.03	42.91–93.16	0	0	0	0	0.82
EORTC QLQ‐BR23								
Systemic therapy side‐effects	0–100	0–57.14	0–57.14	1.5	1.8	0	0	0.43
Arm symptoms	0–100	0–100	0–100	16.4^a^	17.5^a^	1.5	1.8	0.64
Breast symptoms	0–100	0–75.00	0–58.33	7.5	17.5^a^	0	0	0.68
Body image	0–100	0–91.67	0–100	23.9^a^	12.3^a^	0	7.0	0.86
Sexual functioning	0–100	0–83.33	0–100	50.7^a^	59.6^a^	0	1.8	0.77
Future perspective	0–100	0–66.67	0–100	67.2^a^	36.8^a^	0	3.5	N/A
Upset by hair loss (item 35)^b^	0–100	0–100	0–66.67	16.7^a^	11.1	25.0^a^	0	N/A
Sexual enjoyment (item 46)^b^	0–100	0–100	0–100	28.6^a^	15.0	7.1	10	N/A

*Note:* a = Cronbach's alpha.

^a^ = significant floor/ceiling effects (> 15%).

^b^ Optional items: Upset by hair loss T0 *N* = 12, T1 *N* = 9; Sexual enjoyment, T0 *N* = 28, T1 *N* = 20.

Abbreviations: CDRISC, Connor Davidson Resilience Scale; EORTC QLQ‐30, European Organisation for Research and Treatment of Cancer Quality of Life Questionnaire for Cancer Patients; EORTC QLQ‐BR23, European Organisation for Research and Treatment of Cancer Quality of Life Questionnaire for Breast Cancer Patients; GAD‐7, Generalised Anxiety Disorder Scale; PHQ‐9, Patient Health Questionnaire‐9; PHQ‐ADS, Patient Health Questionnaire—Anxiety and Depression Scale.

### 3.6 Outcomes

Table 4 presents scores for interpretation bias measures and estimates of treatment effect on self‐report measures at each time point, adjusted for baseline scores. Regression assumptions, checked using Q‐Q plots and Residual‐Predicted plots, were met. Between‐group differences in interpretation bias, assessed by SST and RT at T1, adjusted for baseline interpretation bias, demonstrated moderate effects in favour of CBM‐I. Treatment effects, based on 95%CIs, were consistent with a range of true effects from either small to very large, in favour of CBM‐I. The upper bound of the 95%CI of Hedge's *g* was above the prespecified progression value (0.5), indicating potential for clinically meaningful benefit.

**TABLE 4:** Means of outcome measures at each assessment and treatment effects.

		CBM‐I		Control		Adjusted mean difference		
Outcome measure	Time	*N*	Mean (SD)	*N*	Mean (SD)	Difference (SE)	95% CI for difference	Hedge's g (95% CI)
SST	0	35	0.65 (0.20)	32	0.61 (0.17)			
	1	28	0.79 (0.17)	29	0.65 (0.18)	0.13 (0.04)	0.047 to 0.204	0.725 (0.195, 1.25)
RT	0	35	−0.50 (0.60)	32	−0.43 (0.56)			
	1	28	0.60 (0.93)	29	0.09 (0.62)	0.53 (0.20)	0.125 to 0.933	0.662 (0.136, 1.19)
CDRISC	0	35	54.2 (9.5)	32	54.3 (12.5)			
	1	28	64.3 (13.9)	29	56.6 (13.7)	8.66 (2.16)	4.37 to 12.9	0.636 (0.110, 1.16)
	2	24	63.1 (13.6)	28	56.1 (13.5)	7.86 (2.25)	3.41 to 12.3	0.577 (0.054, 1.10)
	3	24	63.4 (14.6)	26	57.6 (14.0)	6.73 (2.28)	2.22 to 11.2	0.494 (−0.026, 1.01)
PHQ‐9	0	35	9.4 (4.4)	32	9.4 (4.1)			
	1	28	7.5 (4.4)	29	8.3 (3.9)	−0.80 (0.83)	−2.44 to 0.852	−0.187 (−0.700, 0.326)
	2	24	6.8 (4.7)	28	8.0 (4.0)	−1.32 (0.86)	−3.02 to 0.388	−0.309 (−0.825, 0.206)
	3	24	6.0 (4.5)	26	6.9 (3.7)	−0.85 (0.87)	−2.57 to 0.874	−0.200 (−0.713, 0.314)
GAD‐7	0	35	9.5 (4.2)	32	9.5 (4.8)			
	1	28	6.4 (4.4)	29	7.8 (4.0)	−1.42 (0.98)	−3.35 to 0.519	−0.324 (−0.838, 0.193)
	2	24	6.2 (4.4)	28	7.8 (4.3)	−1.91 (1.01)	−3.92 to 0.089	−0.436 (−0.954, 0.082)
	3	24	6.5 (5.2)	26	7.2 (3.9)	−0.80 (1.02)	−2.82 to 1.23	−0.181 (−0.694, 0.333)
PHQ‐ADS	0	35	18.9 (7.7)	32	18.9 (7.7)			
	1	28	13.8 (8.0)	29	15.9 (7.4)	−2.17 (1.61)	−5.36 to 1.01	−0.278 (−0.793, 0.236)
	2	24	12.8 (8.2)	28	15.6 (7.3)	−3.26 (1.66)	−6.55 to 0.027	−0.418 (−0.936, 0.100)
	3	24	12.4 (8.6)	25	14.0 (7.1)	−1.56 (1.69)	−4.90 to 1.79	−0.200 (−0.714, 0.313)
EORTC QLQ‐30	0	35	68.39 (13.87)	32	68.29 (14.00)			
	1	28	74.21 (13.35)	29	72.26 (11.78)	2.42 (2.05)	−1.64 to 6.47	0.201 (−0.312, 0.715)
	2	24	76.14 (12.17)	28	76.64 (10.75)	−0.528 (2.13)	−4.74 to 3.68	−0.044 (−0.556, 0.468)
	3	24	77.22 (13.23)	25	77.92 (9.20)	−0.535 (2.17)	−4.83 to 3.76	−0.045 (−0.557, 0.468)

Abbreviations: CBM‐I, Cognitive Bias Modification for Interpretation; CDRISC, Connor Davidson Resilience Scale; CI, Confidence Interval; EORTC QLQ‐30, European Organisation for Research and Treatment of Cancer Quality of Life Questionnaire for Cancer Patients; GAD‐7, Generalised Anxiety Disorder Scale; PHQ‐9, Patient Health Questionnaire‐9; PHQ‐ADS, Patient Health Questionnaire–Anxiety and Depression Scale; RT, Recognition Test; SD, Standard Deviation; SE, Standard Error; SST, Scrambled Sentences Task.

On the CD‐RISC, between group differences, adjusted for baseline resilience, demonstrated a moderate effect in favour of CBM‐I, with a trend for the magnitude of this effect reducing slightly over time. Hedge's *g* 95%CIs were consistent with no effect to a large treatment effect in favour of CBM‐I. Between group differences on mood measures (PHQ‐9, GAD‐7 and PHQ‐ADS), adjusted for baseline values, were in favour of CBM‐I, though of a small magnitude, with each showing the greatest difference at T2. Hedge's *g* 95%CIs were consistent with a large treatment effect in favour of CBM‐I to a small risk of harm. There was also a small difference in QoL scores between groups at T1, favouring CBM‐I. This effect was not present at T2 or T3. Hedge's *g* 95%CIs were consistent with a moderate treatment effect in favour of CBM‐I to a moderate risk of harm. Results are similar for additional sensitivity analysis ( Supporting Information S1: Table S4).

### 3.7 Adverse Events and Intercurrent Events

No mental health related serious adverse events (suicide attempts, death or hospitalisations) or intervention‐related adverse events were reported. Post‐randomisation intercurrent events were negative life events reported by 25 participants (control = 13; CBM‐I = 12) and starting psychological or psychiatric intervention in 10 participants (control = 9; CBM‐I = 1). See Supporting Information S1: Table S5.

## 4 Discussion

This study aimed to evaluate the acceptability of a new CBM‐I intervention targeting resilience for women after active treatment for primary BC and determine the feasibility of a full‐scale randomised controlled trial. This novel intervention was designed for people living beyond BC, using a rigorous process of material development.

The study met all pre‐specified criteria to progress to an efficacy trial, other than the target recruitment number, which was three under the target of 70 (‘consider progressing’ range). Difficulties with recruitment are common in web‐based psycho‐oncology intervention research [16], with recruitment challenges exacerbated by the Covid‐19 pandemic [39]. The exclusion rate was high relative to other similar studies [40, 41], however the proportion of eligible participants consenting was also high [42].

Retention and adherence were good. Across the total sample, 71.6% were retained to final assessment (criteria > 70%). Most did not provide a reason for drop‐out or cited family or personal health reasons. CBM‐I adherence was 80% (criteria > 70%). Retention and adherence were similar to other CBM‐I studies [42].

According to acceptability measures and exploratory treatment effect sizes, the CBM‐I intervention appeared both acceptable and potentially beneficial. Median T1 logic and usefulness scores were 3 (criteria > 2). There was an upper limit of Hedge's *g* effect size of 1.19 and 1.25 for the RT and SST measures of interpretation bias, respectively (criteria > 0.5), suggesting that FRAME may be efficacious in changing the hypothesised mechanism of action. Regarding potential clinical benefits of CBM‐I, effect sizes indicate that FRAME had a moderate effect on resilience and small effect on mood. No participants experienced adverse events attributable to the intervention.

### 4.1 Clinical Implications

Findings suggest that interpretation bias, associated with resilience after BC treatment [7], is a promising intervention target. This multi‐session digital CBM‐I programme, adapted from previous studies [24, 25], and tailored to concerns pertinent to BC survivors, was acceptable to participants. A range of treatment effects, as indicated by the pattern of effect sizes and 95% CIs, supports the anticipated mechanism of action, supporting the need for confirmation in an efficacy trial. The FRAME intervention is easy to administer, time‐efficient and easily scalable. If found to be efficacious in a larger trial, FRAME may help meet the needs of BC survivors, by providing an evidence‐based, cost‐effective, accessible intervention.

### 4.2 Study Limitations

Restrictions due to the Covid‐19 pandemic impacted recruitment, as it was not possible to advertise at community events. The pandemic also potentially impacted retention and adherence, for example due to increased caring responsibilities and reduced social support.

The sample were mostly white British, impacting the ability to generalise findings to individuals from other ethnic groups. A significant proportion (*N* = 19) were taking psychotropic medication, and/or currently receiving psychological support (*N* = 18). However, this was required to be stable for 6 weeks prior to taking part, and current treatments appeared well balanced between conditions. Furthermore, only one participant in the CBM‐I group started new treatment during the study, compared with nine participants in the control group. Thus, if anything, this may have reduced the treatment effects observed.

### 4.3 Recommendations

Before a full‐scale trial, researchers should consider altering inclusion criteria and streamlining screening, to ensure the feasibility of large‐scale recruitment. Common reasons for exclusion were not meeting GAD‐7 and PHQ‐9 criteria and completing treatment > 3 years or < 4 months ago. Relaxing criteria may support recruitment. It is also recommended that recruitment methods are altered, to recruit a more diverse sample, for example by collaborating with healthcare services and community organisations.

Regarding outcome measures, the CDRISC, PHQ‐9, GAD‐7 and PHQ‐ADS were appropriate, with good reliability and no significant floor/ceiling effects. However, reliability of some EORTC QLQ‐30 subscales (cognitive functioning, nausea and vomiting) was low (0.67 and 0.50, respectively), with significant ceiling effects. This was potentially influenced by participants experiencing hormone treatment side‐effects. We recommend future studies report on hormone treatment, to facilitate such interpretation. Many of the EORTC QLQ‐BR23 subscales had questionable reliability and significant floor effects, thus impacting responsiveness. Participants mostly completed active treatment over a year ago, and many items relating to side‐effects and symptoms did not to apply. An alternative QoL measure should be considered [43].

### 4.4 Conclusions

Findings support the acceptability of FRAME to promote resilience in women treated for primary BC and indicate that a full‐scale randomised controlled trial is feasible. Results suggest that the CBM‐I reduces negative interpretations relative to a time‐matched control. Effect size estimates suggest moderate treatment effects on resilience, in favour of FRAME, and small effects on mood. These findings strongly support the need for a full‐scale randomised controlled trial.

## Author Contributions

**Anna V. Cartwright:** data curation, formal analysis, validation, visualization, writing – original draft, writing – review and editing. **Hannah Krzyzanowski:** data curation, investigation, project administration, software. **Rona Moss‐Morris:** conceptualization, funding acquisition, methodology, supervision, writing – review and editing. **Laura Smith:** data curation, investigation, project administration. **Yogini Sawjani:** data curation, investigation, writing – review and editing. **Camilla Böhme Kristensen:** investigation. **Nuvera Mukaty:** investigation. **Sam Norton:** formal analysis, supervision, writing – review and editing. **Jo Armes:** conceptualization, funding acquisition, methodology, writing – review and editing. **Colette R. Hirsch:** conceptualization, funding acquisition, methodology, project administration, supervision, writing – review and editing. All authors read and approved the final version of the manuscript.

## Conflicts of Interest

The authors declare no conflicts of interest.

## Supporting information

Supporting Information S1

## Data Availability

The data that support the findings of this study are available from the corresponding author upon reasonable request.
