# Supplementary material for: Feasibility Randomised Controlled Trial of a New Digital Intervention (‘FRAME’) to Promote Resilience in Women Treated for Primary Breast Cancer
Source: Psychooncology. 2025 Jul 9;34(7):e70217. doi: 10.1002/pon.70217 (PMC12240892; doi:10.1002/pon.70217)
Supplement: Supplementary file 1 — Supporting Information S1 [file PON-34-e70217-s001.docx]

Supplementary Materials

**Supplementary Materials 1.**

**Pre-Specified Progression Criteria**

Criteria developed prior to analysis. Where the outcomes fall between ‘progress’ and ‘do not progress’ criteria, the researchers will consider the findings and whether the study was carried out as intended. If there are issues with the intervention, the researchers will consult with experts by experience to further optimise the intervention.

| **Table S1.** Pre-specified Progression Criteria to a Full-Scale Efficacy Trial | | | |
| --- | --- | --- | --- |
| **Measure** | **Progress (‘Green’)** | **Consider progressing (‘Amber’)** | **Do not progress (‘Red’)** |
| Recruitment (% of target sample size) | 100% | 70-100% | <70% |
| Retention to T3 (% retained) | >70% | 50-70% | <50% |
| Intervention Adherence | >70% | 50-70% | <50% |
| Acceptability (average scores) | T1 logic >2  T1 usefulness >2 | T1 logic 1.5-2  T1 usefulness 1.5-2 | T1 logic <1.5  T1 usefulness <1.5 |
| Mechanism change | Upper limit of Hedge’s *g* effect size > 0.5 | Upper limit of Hedge’s *g* 0.2-0.5 | Upper limit of Hedge’s *g* < 0.2 |
| Serious device-related adverse events | No SAEs attributed to the intervention | Rectifiable SAEs attributed to the intervention | Irremediable SAEs or SUSARs attributed to the intervention |
| Abbreviations: SAE = Serious Adverse Event; SUSAR=Suspected Unexpected Serious Adverse Reaction. | | | |

**Supplementary Materials 2.**

**FRAME Intervention Example Scenarios**

Example 1: Active Cognitive Bias Modification for Interpretation (CBM-I) Condition, positively resolved scenario

For these trials, participants listen to the scenario and vividly imagine themselves as the central character. Following the scenario, they are presented with a comprehension question on the screen, with yes/no response options. Accuracy feedback is provided, to reinforce a positive interpretation of the scenario.

| Scenario text:  An opportunity arises to volunteer as a mentor at your local breast cancer survivorship charity. You think about volunteering and feel that your support might be valued. |
| --- |
| Comprehension question:  Could you offer support to someone else?  (correct answer: yes) |

Example 2: CBM-I Condition, ambiguous scenario for participant to resolve

For these trials, participants listen to the scenario and vividly imagine themselves as the central character. Following the scenario, they are presented with a comprehension question on the screen, with yes/no response options. These questions relate to the resolution of ambiguity. Accuracy feedback is provided for correct responses only, to reinforce a positive interpretation of the scenario.

| Scenario text:  A colleague from your work gets in touch to find out how you're doing. They ask when you will be coming back. You tell them it will be a while longer. From the tone of their voice you can tell what they think. |
| --- |
| Comprehension question:  Does your colleague think badly of you for taking time to come back?  (correct answer: no) |

Example 3: Control condition, with factual question

For these trials, control participants listen to the scenario and vividly imagine themselves as the central character. Following the scenario, they are presented with a factual question on the screen, with yes/no response options. Accuracy feedback is provided for incorrect responses.

| Scenario text:  A colleague from your work gets in touch to find out how you're doing. They ask when you will be coming back. You tell them it will be a while longer. From the tone of their voice you can tell what they think. |
| --- |
| Factual question:  Did you say you will go back to work tomorrow?  (correct answer: no) |

Example 4: Control condition, with comprehension question

For these trials, control participants listen to the scenario and vividly imagine themselves as the central character. Following the scenario, they are presented with a comprehension question on the screen, relating to the resolution of ambiguity, with yes/no response options. No feedback is provided.

| Scenario text:  You speak on the phone with a family member you haven't seen for a while. They say your voice sounds weak. You know why this is. |
| --- |
| Comprehension question:  Is your voice weak because you are ill?  (correct answer: no) |

**Supplementary Materials 3.**

**Expectancy and Acceptability Measures**

Expectancy

At the T0 assessment (before the first intervention assignment), participants were asked to rate ‘At this point, how logical does the programme offered to you seem?’ and ‘How useful do you think this programme will be in reducing your level of worry / low mood?’ on 5-point ordinal response scales from 0 ‘not at all logical/useful’ to 4 ‘very logical/useful’. Responses are presented in Table S2.

| **Table S2.** T0 Expectancy Ratings | | | | | |
| --- | --- | --- | --- | --- | --- |
|  | At this point, how logical does the program offered to you seem? N (%) | | | | |
|  | 0  not at all logical | 1 | 2 | 3 | 4  Very logical |
| CBM-I (N=35) | 0 (0) | 0 (0) | 7 (20.0) | 19 (54.3) | 9 (25.7) |
| Control (N=32) | 0 (0) | 2 (6.3) | 8 (25.0) | 15 (46.9) | 7 (21.9) |
|  | How useful do you think this program will be in reducing your level of worry / low mood? N (%) | | | | |
|  | 0  not at all useful | 1 | 2 | 3 | 4  Very useful |
| CBM-I (N=35) | 0 (0) | 0 (0) | 13 (37.1) | 15 (42.9) | 7 (20.0) |
| Control (N=32) | 0 (0) | 2 (6.3) | 17 (53.1) | 10 (31.3) | 3 (9.4) |
| *Note:* CBM-I = Cognitive Bias Modification for Interpretation | | | | | |

Acceptability

At T1, participants were asked to rate ‘After having completed the programme, how logical was the programme offered to you?’ and ‘How useful was this programme in reducing your level of worry / rumination?^[[1]](#footnote-1)^’ on a 5-point ordinal response scale, from 0 ‘not at all logical/useful’ to 4 ‘very logical/useful’. Participants were asked ‘with what degree of confidence would you recommend this programme to a friend with the same level of worry/rumination as you have?^*^’, on a 5-point ordinal response scale from 0 ‘not at all confident’ to 4 ‘extremely confident’. Responses are presented in Table S3.

| **Table S3.** T1 Acceptability Ratings | | | | | |
| --- | --- | --- | --- | --- | --- |
|  | After having completed the programme, how logical was the programme offered to you? N (%) | | | | |
|  | 0  not at all logical | 1 | 2 | 3 | 4  Very logical |
| CBM-I (N=31) | 1 (3.2) | 1 (3.2) | 8 (25.8) | 10 (32.3) | 11 (35.5) |
| Control (N=29) | 2 (6.9) | 6 (20.7) | 5 (17.2) | 8 (27.6) | 8 (27.6) |
|  | How useful was this programme in reducing your level of worry / rumination?  N (%) | | | | |
|  | 0  not at all useful | 1 | 2 | 3 | 4  Very useful |
| CBM-I (N=31) | 1 (3.2) | 6 (19.4) | 6 (19.4) | 15 (48.4) | 3 (9.7) |
| Control (N=29) | 3 (10.3) | 16 (55.2) | 7 (24.1) | 2 (6.9) | 1 (3.4) |
|  | With what degree of confidence would you recommend this programme to a friend with the same level of worry / rumination as you have? N (%) | | | | |
|  | 0  not at all confident | 1 | 2 | 3 | 4  Extremely confident |
| CBM-I (N=31) | 3 (9.7) | 5 (16.1) | 6 (19.4) | 10 (32.3) | 7 (22.6) |
| Control (N=29) | 8 (27.6) | 9 (31.0) | 8 (27.6) | 2 (6.9) | 2 (6.9) |
| Abbreviations: CBM-I = Cognitive Bias Modification for Interpretation | | | | | |

| **Table S4.** Means (SD) of Outcome Measures at Each Assessment and Treatment Effects (Additional Sensitivity Analysis^†^) | | | | | | | | |
| --- | --- | --- | --- | --- | --- | --- | --- | --- |
|  |  | CBM-I | | Control | | Adjusted mean difference | | |
| Outcome Measure | Time | *N* | Mean (SD) | *N* | Mean (SD) | Difference (SE) | 95% CI for difference | Hedge's g (95% CI) |
| SST | 0 | 28 | 0.65 (0.21) | 25 | 0.61 (0.16) |  |  |  |
|  | 1 | 28 | 0.79 (0.17) | 25 | 0.65 (0.18) | 0.11 (0.04) | 0.031 to 0.197 | 0.660 (0.114, 1.21) |
| RT | 0 | 28 | -0.49 (0.63) | 25 | -0.48 (0.52) |  |  |  |
|  | 1 | 28 | 0.60 (0.93) | 25 | 0.09 (0.64) | 0.51 (0.21) | 0.079 to 0.936 | 0.621 (0.077, 1.17) |
| CDRISC | 0 | 28 | 53.8 (9.8) | 25 | 55.2 (12.1) |  |  |  |
|  | 1 | 28 | 64.3 (12.3) | 25 | 57.4 (13.7) | 8.12 (2.23) | 3.70 to 12.5 | 0.600 (0.056, 1.14) |
|  | 2 | 24 | 63.1 (13.6) | 24 | 57.0 (13.6) | 7.18 (2.32) | 2.59 to 11.8 | 0.530 (-0.010, 1.07) |
|  | 3 | 24 | 63.4 (14.6) | 23 | 57.9 (13.4) | 5.87 (2.33) | 1.25 to 10.5 | 0.434 (-0.104, 0.971) |
| PHQ-9 | 0 | 28 | 9.3 (4.4) | 25 | 9.4 (4.1) |  |  |  |
|  | 1 | 28 | 7.5 (4.4) | 25 | 8.6 (3.9) | -1.04 (0.80) | -2.64 to 0.549 | -0.242 (-0.775, 0.292) |
|  | 2 | 24 | 6.8 (4.7) | 24 | 7.7 (4.1) | -0.94 (0.83) | -2.59 to 0.710 | -0.217 (-0.750, 0.316) |
|  | 3 | 24 | 6.0 (4.5) | 24 | 6.6 (3.7) | -0.42 (0.84) | -2.08 to 1.24 | -0.096 (-0.628, 0.435) |
| GAD-7 | 0 | 28 | 10.0 (4.5) | 25 | 9.8 (4.9) |  |  |  |
|  | 1 | 28 | 6.4 (4.4) | 25 | 8.2 (4.1) | -1.85 (1.00) | -3.82 to 0.131 | -0.421 (-0.958, 0.117) |
|  | 2 | 24 | 6.2 (4.4) | 24 | 7.5 (3.9) | -1.59 (1.03) | -3.63 to 0.444 | -0.364 (-0.899, 0.172) |
|  | 3 | 24 | 6.5 (5.2) | 23 | 7.1 (3.8) | -0.62 (1.03) | -2.67 to 1.43 | -0.141 (-0.673, 0.391) |
| PHQ-ADS | 0 | 28 | 19.3 (8.1) | 25 | 19.3 (7.9) |  |  |  |
|  | 1 | 28 | 13.8 (8.0) | 25 | 16.7 (7.6) | -2.84 (1.59) | -6.00 to 0.316 | -0.363 (-0.899, 0.173) |
|  | 2 | 24 | 12.8 (8.2) | 24 | 15.0 (6.8) | -2.55 (1.64) | -5.80 to 0.696 | -0.326 (-0.861, 0.209) |
|  | 3 | 24 | 12.4 (8.6) | 23 | 13.6 (7.1) | -0.94 (1.66) | -4.23 to 2.35 | -0.120 (-0.652, 0.412) |
| EORTC | 0 | 28 | 68.68 (14.35) | 25 | 68.94 (13.40) |  |  |  |
| QLQ-30 | 1 | 28 | 74.21 (13.35) | 25 | 71.70 (12.36) | 2.68 (2.09) | -1.47 to 6.83 | 0.218 (-0.315, 0.751) |
|  | 2 | 24 | 76.14 (12.17) | 24 | 76.40 (11.12) | -0.626 (2.18) | -4.94 to 3.69 | -0.051 (-0.582, 0.481) |
|  | 3 | 24 | 77.22 (13.23) | 23 | 77.63 (9.63) | -0.551 (2.22) | -4.94 to 3.84 | -0.045 (-0.576, 0.487) |

*Note:* ^†^This sample is defined as those who completed at least 8-10 assignments (a full dose) and completed assessments at both T0 and T1 (N=53; 76.8% of total sample).

Abbreviations: CBM-I=Cognitive Bias Modification for Interpretation; SST=Scrambled Sentences Task; RT=Recognition Test; CDRISC=Connor Davidson Resilience Scale; PHQ-9=Patient Health Questionnaire-9; GAD-7=Generalised Anxiety Disorder Scale; PHQ-ADS= Patient Health Questionnaire – Anxiety and Depression Scale; EORTC QLQ-30= European Organisation for Research and Treatment of Cancer Quality of Life Questionnaire for Cancer Patients.

| **Table S5.** Other Treatments Started and Negative Life Events Reported During the Study | | |
| --- | --- | --- |
|  | CBM-I (*n*) | Control (*n*) |
| Other psychological or psychiatric treatments started |  |  |
| Counselling | 0 | 5 |
| CBT | 1 | 2 |
| Psychology Sessions | 0 | 1 |
| SSRI | 0 | 1 |
| Category of negative life event |  |  |
| Physical health problem | 4 | 2 |
| Bereavement | 1 | 3 |
| Investigations for breast cancer | 1 | 0 |
| Friend/family member unwell | 1 | 3 |
| Anniversary of mother’s death | 0 | 1 |
| Relationship difficulties | 2 | 0 |
| Discrimination | 0 | 1 |
| Left job due to bullying | 0 | 1 |
| Covid restrictions impacting social support | 0 | 1 |
| Homeschooling due to Covid | 1 | 0 |
| Damage to car by thieves | 1 | 0 |
| Death of pet | 0 | 1 |
| Financial stress | 1 | 0 |
| Abbreviations: CBM-I=Cognitive Bias Modification for Interpretation; CBT=Cognitive Behavioural Therapy; SSRI=Selective Serotonin Reuptake Inhibitor. | | |

1. Note: The wording of these questions would more appropriately have asked about worry/low mood rather than worry/rumination. [↑](#footnote-ref-1)
